# Highly Enhanced Curcumin Delivery Applying Association Type Nanostructures of Block Copolymers, Cyclodextrins and Polycyclodextrins

**DOI:** 10.3390/polym12092167

**Published:** 2020-09-22

**Authors:** Nóra Zsuzsanna Nagy, Zoltán Varga, Judith Mihály, Attila Domján, Éva Fenyvesi, Éva Kiss

**Affiliations:** 1 Laboratory of Interfaces and Nanostructures, Institute of Chemistry, Eötvös Loránd University, Budapest 112, PO Box 32, H-1518 Budapest, Hungary; nagy.nora.zs@gmail.com; 2 Biological Nanochemistry Research Group, Institute of Materials and EnvironmentalChemistry, Research Centre for Natural Sciences, Magyar tudósok körútja 2, H-1117 Budapest, Hungary; varga.zoltan@ttk.mta.hu (Z.V.); mihaly.judith@ttk.mta.hu (J.M.); 3 NMR Research Laboratory, Instrumentation Center, Research Centre for Natural Sciences, Magyar tudósok körútja 2, H-1117 Budapest, Hungary; domjan.attila@ttk.mta.hu; 4 CycloLab Cyclodextrin Research and Development Laboratory Ltd., Illatos út 7, H-1097 Budapest, Hungary; fenyvesi.e@cyclolab.hu

**Keywords:** curcumin solubilization, Pluronic micelle, cyclodextrin and polymeric cyclodextrin, release of curcumin from drug delivery system

## Abstract

The limited bioavailability of the highly hydrophobic natural compound, curcumin with wide range of beneficial bioactivity is still a challenge. Self-association type systems of polyethylene oxide-polypropylene oxide-polyethylene oxide block copolymers (Pluronic) were applied to enhance the aqueous solubility of curcumin. Comparison of four Pluronics (94, 105, 127,108) with different compositions led to the conclusion that solubilization capacity is maximum for Pluronic 105 with intermediate polarity (hydrophilic/lipophilic balance (HLB) = 15) possessing the optimum balance between capacity of hydrophobic core of the micelle and hydrophilic stabilizing shell of the associate. Curcumin concentration in aqueous solution was managed to increase 105 times up to 1–3 g/L applying Pluronic at 0.01 mol/L. Formation of a host–guest complex of cyclodextrin as another way of increasing the curcumin solubility was also tested. Comparing the(2-hydroxypropyl)-α, β and γ cyclodextrins (CD) with 6, 7 and 8 sugar units and their polymers (poly-α-CD, poly-β-CD, poly-γ-CD) the γ-CD with the largest cavity found to be the most effective in curcumin encapsulation approaching the g/L range of concentration. The polymer type of the CDs presented prolonged and pH dependent release of curcumin in the gastrointestinal (GI) system modelled by simulated liquids. This retarding effect of polyCD was also shown and can be used for tuning in the combined system of Pluronic micelle and polyCD where the curcumin release was slower than from the micelle.

## 1. Introduction

Curcumin is a well-known traditional medicine with wide scale of beneficial bioactivity from powerful anti-inflammatory effect to remarkable antioxidant property [1]. Extensive research over the last half century has revealed several important functions of curcumin. By modulating the activation of various transcription factors, curcumin regulates the expression of inflammatory enzymes, cytokines, adhesion molecules, and cell survival proteins. Today, several clinical trials are studying the effect of curcumin on various diseases such as Alzheimer disease, major depressive disorder, different types of cancer prevention and treatment (pancreatic, colon cancer, etc.), reducing the risk of diabetes and various skin diseases, for example psoriasis [2]. It has also a beneficial effect applied in nanofibrous scaffold for chronic wound treatments [3]. However, the very poor aqueous solubility of curcumin (<0.01 mg/L) and low oral bioavailability (1%) imply a restriction in medical application [4]. 

Various techniques and materials can be used to deliver curcumin in a highly dispersed form to improve its bioavailability. Enhanced solubility and bioactivity of curcumin was reported by loading into casein nanocapsules by Pan and co-workers [5]. The concentration of curcumin entrapped in the nanoparticles was obtained in the range of 10^−1^ g/L. The hydrolysis of curcumin (degradation) was controlled by encapsulating it in phospholipid based nanoemulsion [6]. Lysozyme nanoparticles (d = 130 nm) also proved to be suitable carrier to solubilize curcumin giving a concentration of 10^−2^ g/L and providing a protection of the environment sensitive compound [7]. Drug content in the similar range was achieved using a mixed micellar system of poloxamer and D-α-tocopheryl polyethylene glycol 1000 succinate presenting increased therapeutic efficacy of curcumin [8,9]. A pH sensitive micellar system was developed from diblock copolymers to increase curcumin solubility up to 0.35 g/L [10]. Various curcumin-loaded lipid nanocarriers, nanoemulsions and core-shell nanocapsules were developed and compared in the treatment of inflammatory disease [11]. Furthermore, the lipid matrix composition was analyzed on the structure of nanocarrier [12].

Although there are several reports on achieving the increased solubility of lipophilic drugs such as curcumin, the importance of this task and the medical potential of nanoformulated drugs are still in the front of interest demonstrated by some recent reviews [13,14,15,16]. Considering curcumin’s proven chemopreventive and therapeutic potential, its low cost and pharmacological safety make it worthy to fully evaluate its potential in terms of optimal dose, route of administration, and therapeutic efficacy [1].

Among the nanostructured drug carrier systems the micelles of triblock copolymer with PEO-PPO-PEO structure (PEO: polyethylene oxide, PPO: polypropylene oxide) possess several benefits including the spontaneous formation, and small size (20–60 nm). The biocompatible Pluronics are commercially available in large variety of chemical composition and hydrophilic/hydrophobic character. A further advantageous feature is the much lower critical micellar concentration (cmc) than that of traditional surfactants [17,18], in addition to the long durationstability in reducing the concentration below the cmc [19]. Moreover, some Pluronics show bioactivity preventing the development of multi drug resistance in vitro and in vivo [20,21]. Especially Pluronics with HLB (hydrophilic-lipophilic balance) below 20 are effective in sensitization and inhibition of transporter’s activity of cancerous cells. These properties make Pluronics attractive nanocarriers with a great potential.

Pluronic micelles were used for curcumin encapsulation for drug delivery for the first time by Sahu [22]. The two commonly used and most hydrophilic Pluronics, F127 and F68 were selected for the formulation achieving drug loading in the range of 2–5% w/w. Mixing different Pluronics (F68 and P123) for the solubilization resulted in higher curcumin content, 7% w/w [23], although such binary system besides the enhanced solubilization capacity is described as thermodynamically unstable which finally separates [24,25].

Our aim was to systematically investigate various Pluronics and reveal the effect of composition on their curcumin solubilization ability in the present work. Instead of the frequently used thin-film hydration method applying organic solution we directly solubilized the solid curcumin resulting in saturation condition at each Pluronic concentration up to the gel point. This way the maximum curcumin concentration in water is facilely determined and compared for the Pluronics with various hydrophilic-lipophilic characters (Pluronic 94, 105, 108, 127).

Improving the solubility of hydrophobic molecules through formation of a molecular complex with cyclodextrins has also gained a broad spectrum of applications in diverse fields of drug delivery [16]. Cyclodextrins and their derivatives are biodegradable and non-toxic carbohydrates [26]. Complexation of curcumin was thoroughly characterized by Tonnesen and co-workers [27] presenting increased solubility of curcumin (0.04 g/L) in the form of hydroxypropyl-β-cyclodextrin 1:1 molar ratio complex. The effect of cavity size of various cyclodextrins on the solubility and fluorescence of curcumin were studied by Baglole and co-workers [28]. According to these results solubility of curcumin increased forming 2:1 host:guest complexes with hydroxypropyl-γ-cyclodextrin as the best solubilizer. From the decrease of fluorescence intensity, a folded structure of curcumin in this complex was presumed. Introduction of polymeric β-cyclodextrin, in addition to the improved solubility of lipophilic drugs by inclusion complex formation, is expected to allow the control of sustained release [29]. Although the degree of increased solubility of curcumin reported in previous works is notable, the results are somewhat controversial, considering the composition, stability as well as the influence of cyclodextrin structure. The preparation technique of complexes furthermore seems also to affect the degree of solubilization. There is a lack of systematic assessment including various types of cyclodextrins and characterization of the molecular interactions in their inclusion complex with curcumin.

Therefore highly water soluble cyclodextrin derivatives with various cavity size (α, β, γ) and their polymeric versions (poly-α-CD, poly-β-CD and poly-γ-CD) were used in our comparative study to enhance the curcumin solubility. The structure of the complex, the molecular interaction between the host and guest components in addition to the drug loading and release were analysed facilitating the selection of the most potent delivery system. The evaluation of the properties of the curcumin containing associated systems allowed finding the criteria for the optimum structure.

Based on the results a trial was made to combine Pluronic micellar system with polymeric CD derivative. According to our knowledge that is the first time to use polyCD in controlling of curcumin release from polymeric micellar carrier.

## 2. Materials and Methods

### 2.1. Materials

Curcumin (M_w_ = 368.38 g/mol; purity ≥ 65% by HPLC), the poly(ethylene glycol)-*block*-poly(propylene glycol)-*block*-poly(ethylene glycol) block copolymers, Pluronic F108 and F127 were purchased from Sigma-Aldrich (St. Louis, Missouri, USA), P94 and P105 were a kind gift of BASF Hungary(Budapest, Hungary). Main properties of the Pluronics, structure, critical micellar concentration (cmc), hydrophilic/lipophilic balance (HLB) are summarized in Table 1. The 2-hydroxypropyl-α-cyclodextrin (HP-α-CD), 2-hydroxypropyl-β-cyclodextrin (HP-β-CD), 2-hydroxypropyl-γ-cyclodextrin (HP-γ-CD) and the soluble α/β/γ-cyclodextrin polymer crosslinked with epichlorohydrin (poly-α-CD/poly-β-CD/poly-γ-CD) were received from Cyclolab Ltd. (Budapest, Hungary), used as received. Their main properties are shown in Table 2. Methanol, ethanol, dimethyl-sulfoxide (DMSO) and acetone were of laboratory grade from Molar Chemicals Kft. (Budapest, Hungary) while sodium chloride, sodium hydroxide, potassium dihydrogen phosphate, disodium hydrogen phosphate and sodium dodecylsulfate (SDS) from Sigma-Aldrich (St. Louis, MO, USA) were of analytical (ACS) grade.

**Figure 1:**
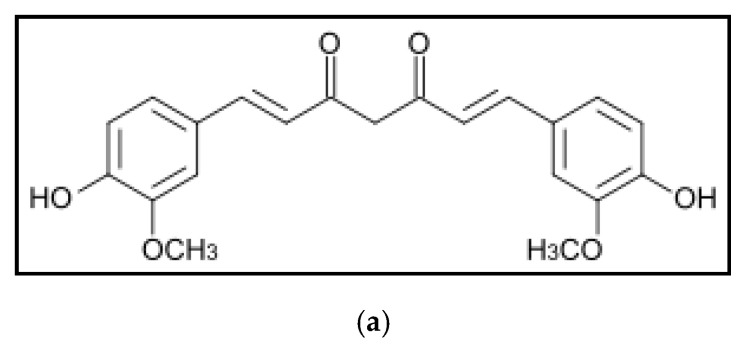

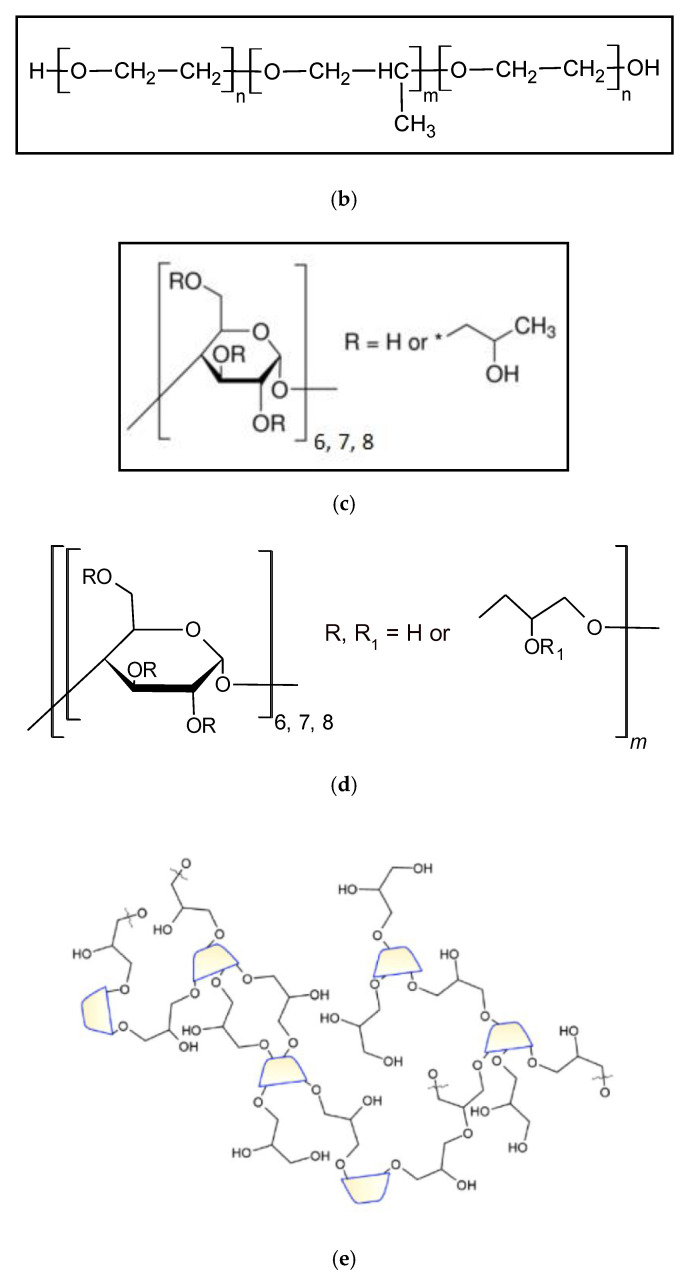
Chemical structure of the curcumin (keto form): (**a**) Pluronics; (**b**) hydroxypropyl-cyclodextrin derivatives; (**c**) polycyclodextrins (**d**,**e**) *m* = 110–140.

The chemical compositions of the curcumin (Figure 1a), block-copolymers (Figure 1b) and cyclodextrin derivatives (Figure 1c–e) are shown below.

### 2.2. Sample Preparation

#### 2.2.1. Preparation of the Curcumin Loaded Pluronic Micelles

The Pluronics have been dissolved in double distilled water at 36.5 ± 0.5 °C and the solid curcumin was added to the solution with continuous stirring. The samples were stirred at 36.5 ± 0.5 °C for 7 days. To remove the excess curcumin the micellar solutions have been filtered through 800, 450 and 200 nm pore size filters subsequently. We found that the saturation is complete after 7 days and further stirring makes no significant difference.

For the cyclodextrinstabilized Pluronic micelles after the filtration of 9 mL micellar solution 1 mL 100 mg/mL polymer-β-cyclodextrin solution has been added and stirred continuously for 24 hours at 36.5 ± 0.5 °C.

Size and size distribution were measured by NanoLab 3D light scattering instrument (LS Instruments AG, Fribourg, Switzerland). Average size with PDI and size distribution using Contin and CURENN analysis were evaluated.

#### 2.2.2. Dissolution of Curcuminin Cyclodextrin Solutions

Calculated amounts of the different types of cyclodextrins have been dissolved in 10 mL water at room temperature. The 0.02 g curcumin was dissolved in 1 mL acetone and rapidly added to the aqueous solution next to continuous stirring. After the acetone has been evaporated from the system the samples have been filtered through 800 and 200 nm pore size filters to eliminate the uncomplexedcurcumin. The initial cyclodextrin:curcumin molar ratio was 2.5:1. For the polymer cyclodextrins we applied double weight of the hydroxypropylcyclodextrin amount as the cyclodextrin content of the polymers is ~50–70% m/m [33].

### 2.3. Characterization of Curcumin Loaded Nanostructures

#### 2.3.1. Drug Content

To determine the drug content, the aqueous samples were either diluted by equal volume of methanol (micellar systems) or dried and redissolved in methanol or DMSO for cyclodextrins and cyclodextrin polymers, respectively. The absorbance was measured spectrophotometrically (Specord 40, Analytik Jena AG, Jena, Germany) at 427 nm. Each measurement was repeated three times. The drug concentration, *c_curc_* (mg/L) was calculated for the aqueous medium of the nanoparticles and presented standard deviation <±4%.

#### 2.3.2. FTIR Spectroscopy

IR spectra (128 scans, 4 cm^−1^ spectral resolution) of the CD-complexes were recorded in attenuated total reflection (ATR) mode using a dynamically aligned Varian 2000 FTIR spectrometer (Varian Inc., Palo Alto, CA, USA) equipped with a liquid nitrogen cooled broadband MCT detector and fitted with a ‘Golden Gate’ single reflection diamond ATR unit (Specac Ltd., Orpington, UK). 5 μL of sample solution was spread on the top of the ATR crystal and a thin dry film was obtained by evaporation of the aqueous solvent under ambient conditions. All spectra were ATR corrected. For spectral manipulations the GRAMS/AI (7.02) software package was used.

#### 2.3.3. NMR Spectroscopy

Solution state NMR spectra were obtained by a Varian NMR system spectrometer (Varian Inc., Palo Alto, CA, USA) operating at the ^1^H frequency of 600 MHz (150 MHz for ^13^C) with a 5 mm indirect detection tuneable triple-broadband probe. The cyclodextrin samples were dried in vacuum at room temperature then dissolved in deuterated water (D_2_O) and measured at 25 °C in all cases. Qualitative ^1^H spectra were recorded 16 s of relaxation delay, 4 second of acquisition time and solvent suppression.

#### 2.3.4. In vitro Drug Release and Release Kinetics

The in vitro curcumin release from the cyclodextrin complexes was investigated into simulated gastrointestinal liquids for 24 h [34,35] for which 3 mL of each sample was transferred into 3500 Da dialysis bag. The bags were suspended into 50 mL release medium which was constantly stirred with 100 rpm and kept at 37 ± 0.5 °C. Samples were withdrawn and replaced with fresh solution at appropriate time intervals from the outer solution to determine the amount of the drug released. To mimic the in vivo curcumin release characteristics from the drug carriers we applied the following time intervals and media:

Stage 1—Simulated gastric fluid: 0–2 h; pH 1.2; 0.2 g NaCl, 0.32 g pepsin, 0.7 mL cc HCl in 100 mL water.

Stage 2—Simulated intestinal fluid: 2–5 h; pH 6.8; 0.68 mg KH_2_PO_4_, 7.7 mL of 0.2 M NaOH in 100 mL water.

Stage 3—Simulated colonic fluid: 5–24 h; pH 7.4; phosphate buffer

The different release media contained 2%SDS to maintain sink condition. The drug content was determined spectrophotometrically.

## 3. Results and Discussion

The aim of this study is to increase water solubility of hydrophobic drug agents by applying biocompatible/biodegradable polymers.

### 3.1. Solubilisation in Pluronic Micelles

Enhanced solubility of curcumin in aqueous medium was planned to achieve by its solubilization in tri-block copolymer (Pluronic) micelles. Pluronics were applied in a wide concentration range (from 10^−5^ to 10^−2^mol/L) above the corresponding critical micellar concentration. The influence of the chemical composition of the Pluronics was also studied comparing the behavior of Pluronic 94, 105, 108 and 127.

**Figure 2:**
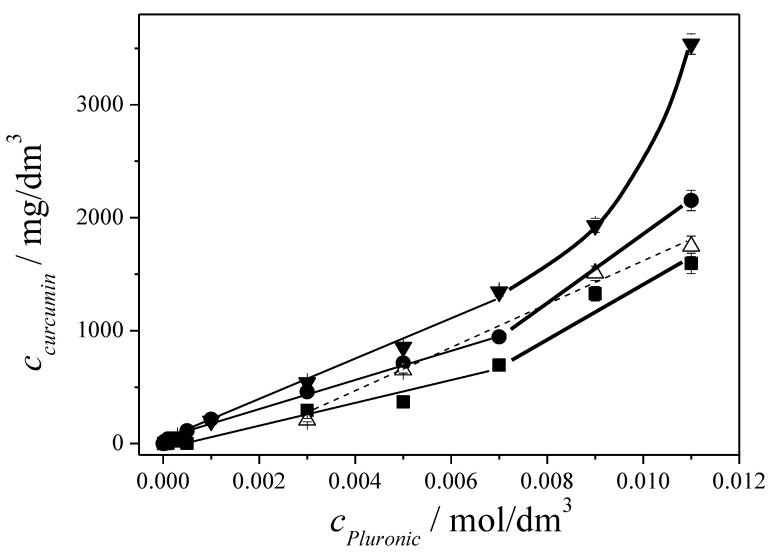
Curcumin concentration as a function of molar concentration of Pluronic 108 (■),94 (△), 127 (●) and 105(▼) in aqueous solution. (Lines are just to guide eyes).

The solubilized concentration of curcumin as a function of molar concentration of the various Pluronics is presented in Figure 2.

Relating to the very low solubility of curcumin in water (<0.01 mg/L) a significant increase was found even at low concentration of the Pluronics. One of the main advantages of the polymeric micellar systems is the low critical micellar concentration, which is in the micromolar range for the Pluronics used in the present work (Table 1). Above that concentration a gradually increasing amount of curcumin is solubilized within the core of the Pluronic micelles resulting in enhancement of curcumin solubility by 5 orders of magnitudes up to 3 g/L.

The composition of the tri-block copolymers has a clear influence on the amount of curcumin solubilized. There is a significant difference in the measured curcumin concentration; the highest amount was obtained for Pluronic 105, while the Pluronic 108 was the least effective, with the Pluronic 94 and 127 in between them. Considering the concentration dependence of solubilization the expected linear change in the curcumin concentration due to the increasing number of the micelles is seemed to be valid, although from about 0.007 M Pluronic concentration a more pronounced increase is observed for Pluronic 105, 108, and 127. The solubilizationcapacity data (molcurcumin/molPluronic) determined from the slope of the curves is summarized in Table 3.

The solubilization capacity values (*S*) are different presenting the lowest value for Pluronic108 which is the most hydrophilic copolymer with 80% of PEO content while almost double for Pluronic 94 with 40% of PEO, which is the most hydrophobic one. The solubilization capacity values follow the hydrophobicity order expressed also by their HLB values (Table 1). The comparison allows the selection of the most potent block-copolymer for the solubility enhancement of a hydrophobic drug molecule.

For such a hydrophobic molecule as curcumin its location in the polymeric micellar structure is supposed to be mainly the core. Based on this assumption the highest solubilization capacity is expected for a Pluronic with the longest PPO block, which is the 127 in the series studied here however, 105 proved to be more efficient. The reason for that might be the finding that the core of a Pluronic micelle is not completely hydrophobic. Alexandridis and his co-workers [36] reported on probing the micropolarity in the aqueous copolymer solution using the intensity ratio of the pyrene vibrational fine structure in fluorescence emission spectra. The results showed presence of approximately 4% of water and significant (25–30%) PEO content in the micelle core as the copolymer becomes more hydrophilic. Therefore, the incorporation of curcumin molecules into the core of more hydrophilic Pluronic 127 is less favoured than into a core of a micelle formed from a less hydrophilic Pluronic, like Pluronic 105. This points to the importance of the copolymer composition in determining micellar structure, the core’s polarity and hence solubilization properties.

The Pluronic micellar solutions are characterized by a large polydispersity (PDI: 1.1–1.3). That is because the “empty” micellar system shows a bimodal size distribution. The typical size of a Pluronic 105 micelle in the 1–9 mM concentration range is obtained 24–28 nm in diameter. The presence of a significant amount (50–60%) of larger micellar aggregates with a diameter of 250–300 nm is also indicated.

It is interesting to see that the Pluronic 105 systems with solubilized curcumin are characterized by a slightly larger micelles with 30 nm in diameter and lower polydispersity (0.5–0.6). According to the size distribution results most of the micelles are in the individual state and their possible aggregation is highly suppressed. That finding is in line with Alexandridis explanation on the polarity of the core of Pluronic micelle [36]. The highly hydrophobic curcumin increases the hydrophobicity of the core of Pluronic micelle enhancing the orientation of PEO chains towards the aqueous phase thus raising the stability against aggregation. That behavior is a nice example of the influence of a foreign material on the structural behavior of Pluronics in aqueous environment.

The solubilization curves above a certain Pluronic concentration present a marked change in the slope especially for Pluronic 105 and 108 (Figure 2). This increased solubilization might be the indication of some structural change in the self-association of triblock copolymers.

According to the phase behavior of binary (water–Pluronic) systems there is a micellar solution at concentration and temperature range approximately up to 20% w/w and 60 °C [37]. In accordance with that it was concluded that at least 20% or 30% w/w of Pluronics needed in general for micellar gel formation [38]. Since all of our Pluronic concentrations are below this specific concentration the observed change in solubilization capacity is somewhat surprising.

On the other hand, as reported by Ivanova [39] and Jang [40] the presence of a third component, as cosolvent, additive, or the curcumin itself in our case, can greatly modify the phase behavior of the aqueous Pluronic systems. This is supported by our finding that the Pluronic systems with or above 0.01 M concentration containing significant amount of solubilized curcumin showed a gel like appearance, although the Pluronic concentration was well below 20% w/w.

Differences in the fine structure even within the micellar phase range of aqueous Pluronic system were revealed in an earlier work of Rapoport and Caldwell [41]. Micellization of Pluronic 105 solution was investigated by EPR spectroscopy using a spin-labeled stearic acid three different regimes were identified ranging from small aggregation complex, in which the probe was fully surrounded by water, via a regime of larger multimolecular complexes, still with a high penetration of water, to a region of highly compacted multimolecular structures with very low water content. These highly compacted multimolecular micelles with hydrophobic cores had greatly enhanced solubilization efficiency. This transition of the micellar structure of Pluronic 105 was characteristic in the concentration range of 1–10% w/w. This finding could provide an explanation for our results too, namely the highly increased solubilization of curcumin starting from 0.007 M (4.5 w/w) of Pluronic 105. It is reasonable to suppose that similar compact micellar aggregation can lead to the enhanced solubilization ability in the case of the Pluronic 108, although at relatively lower degree.

We can conclude from the comparative evaluation of the curcumin solubilization results that for efficient solubilization by Pluronic copolymers it is important to have a high capacity core (long PPO block) with proper polarity (HLB < 20) and sufficiently thick shell of hydrophilic chains (sufficiently long PEO blocks) for the stabilization in aqueous environment. According to our investigations Pluronic 105 was found to meet these requirements for curcuminsolubilisation providing highly enhanced solubility (3.5 g/) especially applied the block copolymer at high concentration (>5 *w*/*w*%).

### 3.2. Curcumin—Cyclodextrin Derivates Complexes

We applied hydroxypropyl-α/β/γ-cyclodextrins and their polymers to investigate which is the most suitable for the curcumin molecule. At preliminary tests several sample preparation methods have been tried and we found that the highest drug content can be achieved by the method mentioned in Section 2.2.2. The drug-cyclodextrin complexes were investigated in the solution state by NMR and in the solid state (as dry film) by FTIR measurements as the cyclodextrins affect on the absorbance spectra [42,43].

#### 3.2.1. NMR

**Figure 3:**
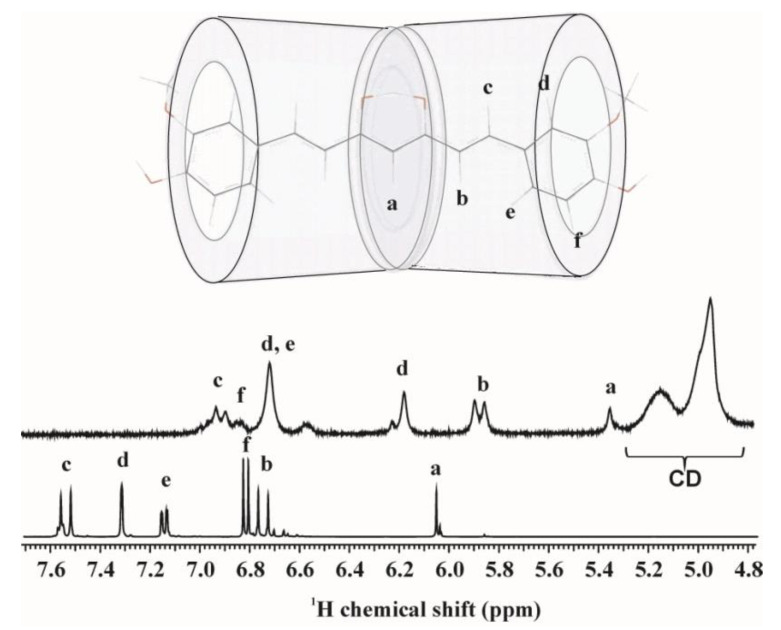
^1^H NMR of curcumin/HP-γ-CD complex in D_2_O (upper spectrum) and curcumin in DMSO-d_6_ (lower spectrum). The schematic CD rings represents the proposed complex structure.

**Figure 4:**
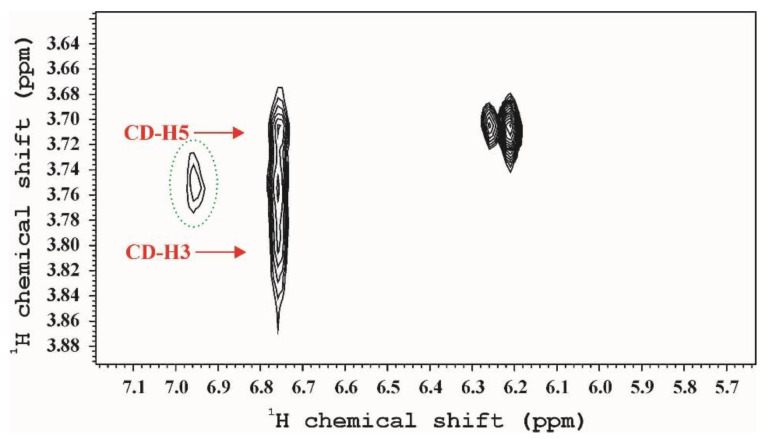
ROESY spectrum of γ-CDcurcumin complex. The green ellipsoid notesthe trivial cross peak between methoxy group and ‘d’ hydrogen of curcumin.

Solution state NMR techniques were applied to study the complex formation leading to increased solubility of curcumin. The measurements allow determining the drug–cyclodextrin ratio and to investigate the structure of the complexes. NMR parameters of curcumin are solvent dependent because of the keto-enol tautomerism and no reference spectrum in aqueous solution exists since its extremely low solubility of water. All the recorded ^1^H spectra differ from each other, but they allow determining the curcumin/cyclodextrin ratio because the delocalized signals of curcumin are separated from the signals of cyclodextrins. The drug-CD ratio is very low for α- and β-CD solutions as Table 4 shows. This ratio should be 0.5 for inclusion complexes, if each aromatic ring is complexed by a CD [44]. On the other hand, the inclusion complexation should result uniform chemical environment for the curcumin molecules and structured NMR spectra. Only the HP-γ-CD complex gave structured ^1^H spectra, as Figure 3 shows. Two dimensional NOESY and ROESY spectraprove the formation of inclusion complexes not only for the monomeric but the polymeric γ-CDs (Figure 4), cross peaks of H3 and H5 hydrogens of γ-CD both with the conjugated chain and aromatic signals clearly show the existence of inclusion complex. Summarizing these observations we can conclude, that complexation of curcumin by CDs is possible on two ways: i. inclusion complexation in case γ-CD and poly-γ-CD (because of the hindered segment motions not all the CDs can form complexes), ii. complexation by outer functional groups of CDs in case of monomeric and polymeric α-, β-CDs.

Polarity of the solvent has a large influence on the chemical shift values (both on ^1^H and ^13^C) and NMR parameters are a great help to differentiate between keto, enol or equilibrating forms. In case of HP-γ-CD the almost full assignation of curcumin was possible. The^1^H and ^13^C chemical shift values clearly show that curcumin exists in the inclusion complex in the equilibrating keto-enol form [45] (the largest chemical shift value on the ^13^C scale is 182.3 ppm) which means that we can close out the folded structure as it was mentioned previously [21]. The ^1^H signals are shifted relative to the chemical shifts measured in DMSO, methanol or CDCl_3_. The chemical shift values of curcumin are shifted with 0.4–1.1 ppm relative to the values detected in DMSO, except with *f* noted aromatic and the methoxy signals which are outside of the CD cage as noted on the Figure 3. These latter signals are shifted only with 0.1 ppm which can be explained by the solvent effect. The larger shift differences suggest that the delocalized electron structure is disturbed by complexation. The NMR spectrum consists from more signal series (for example with *d* noted signal), thus most probably there is no favoured orientation of the CD rings.

#### 3.2.2. Infrared (IR) Spectra of Cyclodextrin—Curcumin Complexes

**Figure 5:**
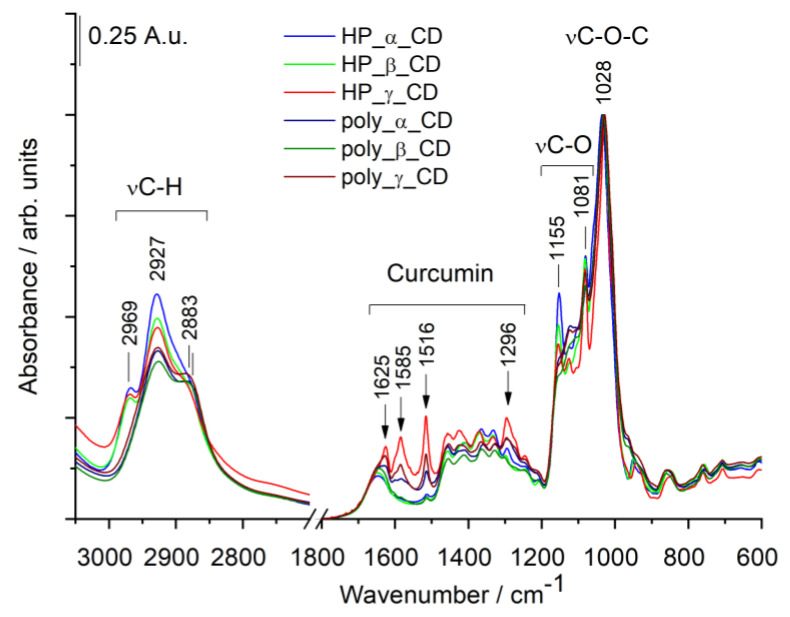
Infrared(IR)spectra of curcumin loaded CD samples. Spectra are normalized to the C-O-C stretching band around 1028 cm^−1.^

Figure 5 shows the IR spectra of curcumin loaded CD samples.

All CD samples exhibit prominent broad IR bands around 3400 cm^−1^ due to −OH stretching vibrations (not shown). In Figure 5 characteristic CD bands at 1028 cm^−1^ and at 1081 and 1155 cm^−1^ are present. The former corresponds to C-O-C stretching (νC-O-C) of glucose ring and glycosidic bonds, while the latter two bands belong to C-O stretching vibrations (νC-O) of −C−OH moieties [46,47]. It is worth to note, that in the spectra of poly-CD samples the intensity of the bands at 1155 cm^−1^ is reduced. The absorption bands at high wavenumbers can be assigned to asymmetrical and symmetrical stretching vibrations of C-H bonds (at 2969, 2927 and 2883 cm^−1^, respectively). The presence of curcumin can be witnessed by the presence of typical curcumin bands at 1625, 1585, 1516 and 1296 cm^−1^ [44,45].We have to point out, however, that in some cases only the most intense curcumin band around1516 cm^−1^, assigned to mixed C=O stretching and CCC and CC=O bending vibrations, can be observed. Based on the band intensities, an assessment of the relative curcumin content can be performed. Using the integrated intensity of the curcumin band around 1516 cm^−1^ (from 1510 cm^−1^ to 1495 cm^−1^) the highest curcumin loading capacity was established for HP-γ-CD. Roughly, only the half amount of drug could be loaded into poly-γ-CD. In case of β- and α-CD samples, lower amount of curcumin was detected (Table 4), in line with the outcome of NMR measurements. We have to point, however, that the amount of encapsulated curcumin assessed by IR method is only an index number and can be used solely for inter-sample comparison.

The encapsulation efficiency of the various CD derivatives, HP-γ-CD>poly-γ-CD>poly-α-CD>poly-β-CD>HP-α-CD>HP-β-CD is in accordance considering both of the measurements.

**Figure 6:**
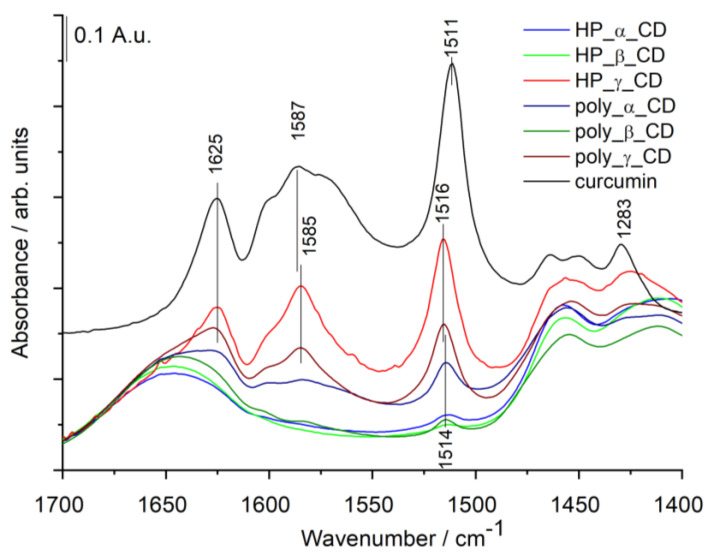
Typical IR absorption bands of curcumin (pure curcumin from ethanol solution and curcumin loaded CD samples).

More interesting are the shifts of curcumin bands compared with that of pure curcumin (measured from ethanol solution). Figure 6 shows the enlarged part of the IR spectra comprising the typical curcumin bands at 1626 cm^−1^ (C=C and C=O stretching of the interring chain), 1587 cm^−1^ (C-C stretching of aromatic rings), 1511 cm^−1^ (combination of C=O stretching and CCC and CC=O bending vibrations) and 1283 cm^−1^ (C-H bending). As to the β- and α-CD samples, the band at 1511 cm^−1^ is shifted to 1514 cm^−1^. The same frequency shift was observed by Mangolim et al. [48] for curcumin-β-CD complex obtained by co-precipitation.

As to the γ-CD samples, a pronounced shift of the 1511 cm^−1^ band towards high wavenumbers was observed attended by the shift of C-C stretching of aromatic rings. Lopez-Tobar et al. [49], by using Raman spectroscopy to study encapsulation of curcumin by cyclodextrins, suggested, that upon encapsulation the phenyl groups are directly involved in the curcumin–CD interaction and due to the changes induced in electron conjunction between C=C and keto-enol groups, an isomerization of inter-chain moiety might happen. We observed similar effects. Compared with the spectrum of pure curcumin dissolved in ethanol, a shift of IR bands related to coupled C=O vibrations towards higher wavenumber occurs for all CD samples. If we postulate, that in ethanol the curcumin exist only in enol form [50], confirmed also by the band positions of conjugated C=O/C=C stretching vibrations [49], we can suggest that a minor isomerization towards keto form upon CD encapsulation might take place. This phenomenon is more significant in case of γ-CDs. Since the same frequency shift was observed for both HP-γ-CD and poly-γ-CD samples, it seems plausible that the curcumin-CD complex formation mechanism might be the same in both cases.

#### 3.2.3. Drug Release

**Figure 7:**
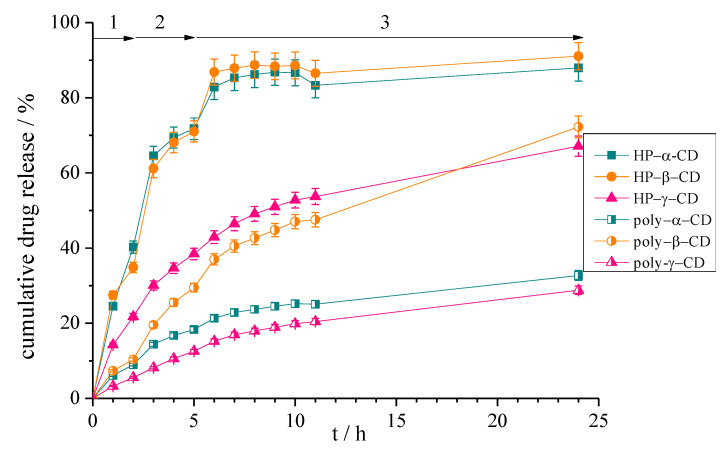
The cumulative curcumin release curves for the different types of cyclodextrin complexes. (Model fluids: 1 simulated gastric fluid, 2 simulated intestinal fluid, 3 colonic fluid).

The drug release tests have been designed to simulate the human digestion tracks. The curves for cumulative drug release from the different cyclodextrin complexes are presented in Figure 7.

From HP-α-CD and HP-β-CD systems high portion of curcumin is released in the 2 hours period in the gastric fluid. Further amounts were observed in the following stage in the intestine fluid, although an additional limited amount is released in the last period. For the other systems the release profile is different. A smaller gradual release was obtained resulting in about 60% for poly-β-CD and HP-γ-CD while about 30% cumulative value was found for poly-α-CD and poly-γ-CD. The last two systems produce a noteworthy retard effect.

**Figure 8:**
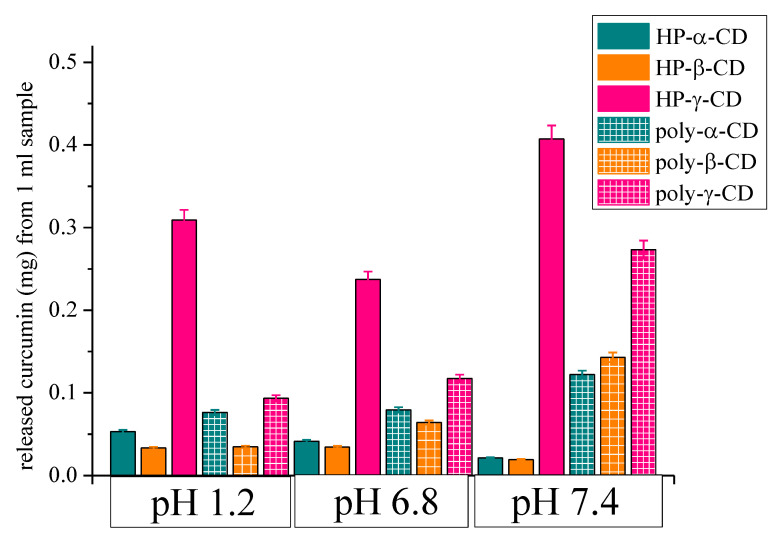
The released amount of curcumin (mg) from 1 mL CD complex sample at three stages of model gastrointestinal tract; pH 1.2, 6.8 and 7.4 corresponds to simulated gastric, intestinal, and colonic fluid, respectively.

Besides the cumulative release expressed as relative amount (%) it is worth to consider and compare the absolute amount of curcumin released from the various CD-derivatives which is summarized in Figure 8. This representation is more informative to find the best system for drug carrier purposes even with the aim of targeted release. Amounts of curcumin released at the three stages of gastrointestinal tract are plotted for the six various CD derivatives. The highest amount of curcumin was released from HP-γ-CD complex in accordance with the most efficient encapsulation of that CD derivative determined by NMR. The largest amount, 0.4 mg was detected in the colonic fluid, while 0.3 mg in the gastric and less amount in the intestinal fluid. For poly-γ-CD complex a significant released amount was also determined, especially in the colonic region. For the other systems the released amount in each stage is below 0.1 mg, except poly-α-CD and poly-β-CD in the colonic fluid. It is interesting to note, that there are systems, HP-α-CD and HP-β-CD, which show increased release in the first and second stages comparing to the colonic region. Such differences allow the design of targeted drug delivery using various CD-derivatives.

### 3.3. Curcumin Loaded Micellar System Stabilized by Poly-β-CD

As it is shown by the previous release results the poly-CD complexes exhibited specific retard effect. With the aim to control the release of curcumin from the polymeric micellar system poly-CD was applied in a combined system. After the identification the Pluronic block-copolymer which can solubilize the highest amount of curcumin, Pluronic 105 micellar system was prepared and further modified with application of poly-β-CD. Micellar and the combined systems have been tested to compare their curcumin release properties.

#### Release from the Micellarand Poly-β-CD Combined Systems

**Figure 9:**
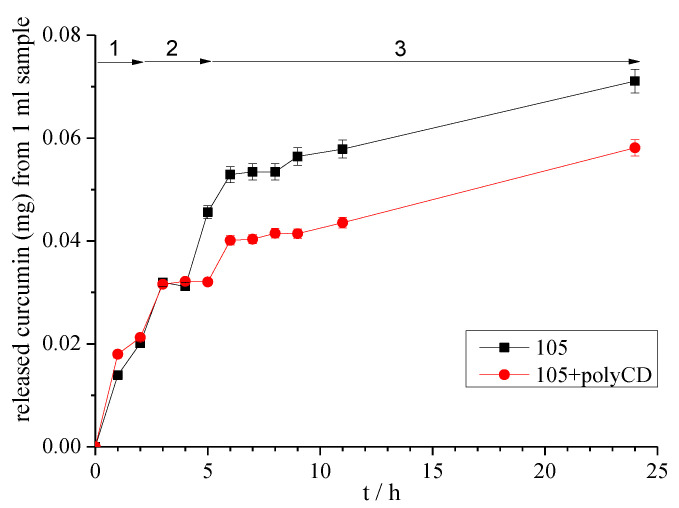
Amount of released curcumin from the Pluronic 105 micellar system and its stabilized form by poly-β-CD (Model fluids: 1 simulated gastric fluid, 2 simulated intestinal fluid, 3 colonic fluid).

The drug release tests have been done for the highest applied Pluronic 105 concentration which is 1.1 × 10^−2^ M. Poly-β-CD was applied following the solubilization procedure. The released amount of curcumin from 1 mL samples as a function of time using the simulated fluids is plotted in Figure 9.

The release pattern shows a gradual change in the studied 24 h period. A certain amount of curcumin is released into each of the model liquids: gastric, intestinal and colonic. The added poly-β-CD however, results in sustained drug release from the Pluronic micellar system which is especially observable after 4 hours. The PEO chains of the Pluronic 105 may form a host:guest complex with the cyclodextrin ring thus slowing the release the of encapsulated curcuminin the core of the micelle [50,51].

This combined system possesses the advantage of high capacity micellar nanocarrier together with the retard effect obtained by the poly-CD adsorption.

## 4. Conclusions

For the nanoencapsulation of hardly soluble curcumin polymeric micellar and various CD-derivative complexes were prepared with the aim of enhanced bioavailability. The introduction of curcumin into such molecular association systems is simple and straightforward resulting in high curcumin concentration in water.

Block-copolymer micellar systems have high solubilization capacity by increasing the Pluronic concentration up to the gelation point. Among the four Pluronics with various structures P105 with medium overall hydrophobicity was found to be the most efficient. Curcumin concentration in water achieved was in the g/L range which means enhancement of the aqueous solubility by 5 orders of magnitude.

The highest curcumin concentration in the form of cyclodextrin complex was similarly about 2 g/L in the case of HP-γ-CD applied. The reason for that is probably the large cavity of γ-CD which seems to favor the inclusion and the high aqueous solubility of the HP-CD-derivative. The polymeric-CD complexes presented sustained curcumin release with various degree in different stages of model gastrointestinal system. This character of the polyCD carriers might be exploited in formation of micellar-polyCD combined system with controlled release as well as targeting curcumin mainly into colonic, gastric or intestinal system.

## Figures and Tables

**Table 1:** Main properties of the applied Pluronics.

Pluronic	*M*/g·mol^−1^	n_PEO_-m_PPO_-n_PEO_	cmc/mol·L^−1^ (37 °C)	cmc/g·L^−1^ (37 °C)	HLB
94	4600	24-47-24	4.3 × 10^−6 a^	0.020	13.5
105	6500	37-56-37	1.5 × 10^−5 b^	0.097	15
108	14,600	132-56-132	2.2 × 10^−5 c^	0.32	27
127	12,600	97-63-97	2.8 × 10^−6 c^	0.034	22

^a^ cmc at 40 °C [30], ^b^ determined from surface tension, ^c^ [31].

**Table 2:** Main properties of the applied cyclodextrin derivatives.

Cyclodextrin	*M*/g·mol^−1^	Inner Diameter of Cavity ^a^/nm	Degree of Substitution
HP-α-CD	1180	0.53	3.6
HP-β-CD	1396	0.65	4.6
HP-γ-CD	1580	0.83	4.8
poly-α-CD	~150,000		5–6 ^b^
poly-β-CD	~165,000		5–6 ^b^
poly-γ-CD	~227,000		5–6 ^b^

^a^ [1,6] The cavity size of the Cyclodextrin units within the polymers are identical with those for the hydroxypropylated derivatives but further cavities formed by the network are also present. ^b^ The degree of substitution values were calculated from the NMR results [32].

**Table 3:** Solubilization capacity in the linear range (*S*) and at Pluronic concentration of11mM (S*) determined for curcuminsolubilization in Pluronicmicellar systems at 37 ± 0.5 °C.

Pluronic	*S*/mol_curcumin_/mol_Pluronic_	S*/mol_curcumin_/mol_Pluronic_
108	0.287	0.39
127	0.362	0.530
105	0.473	0.875
94	0.526	0.526

**Table 4:** Relative amount of curcumin to CD on the base of IR intensity ratio of two characteristic bonds (A (1516 cm^−1^)/A (1027 cm^−1^)) and molar ratio of the complexes determined from NMR.

Sample	IR: Relative Amount of curc/C-O-C bonds (A (1516 cm^−1^)/A (1027 cm^−1^))	NMR:
HP-α-CD	0.12	0.027
HP-β-CD	0.06	0.007
HP-γ-CD	2.44	0.402
poly-α-CD	0.50	0.093
poly-β-CD	0.14	0.088
poly-γ-CD	1.39	0.179
